# Development of a 3D workspace shoulder assessment tool incorporating electromyography and an inertial measurement unit—a preliminary study

**DOI:** 10.1007/s11517-017-1745-4

**Published:** 2017-11-11

**Authors:** Navid Aslani, Siamak Noroozi, Philip Davenport, Richard Hartley, Mihai Dupac, Philip Sewell

**Affiliations:** 1 Department of Design and Engineering, Faculty of Science and Technology, Bournemouth University, Talbot Campus, Fern Barrow, Poole, Dorset BH12 5BB UK; 2 Bournemouth Royal Hospital, Bournemouth, UK

**Keywords:** Shoulder ROM, IMU, EMG, Assessment tool

## Abstract

Traditional shoulder range of movement (ROM) measurement tools suffer from inaccuracy or from long experimental setup times. Recently, it has been demonstrated that relatively low-cost wearable inertial measurement unit (IMU) sensors can overcome many of the limitations of traditional motion tracking systems. The aim of this study is to develop and evaluate a single IMU combined with an electromyography (EMG) sensor to monitor the 3D reachable workspace with simultaneous measurement of deltoid muscle activity across the shoulder ROM. Six volunteer subjects with healthy shoulders and one participant with a ‘frozen’ shoulder were recruited to the study. Arm movement in 3D space was plotted in spherical coordinates while the relative EMG intensity of any arm position is presented graphically. The results showed that there was an average ROM surface area of 27291 ± 538 deg^2^ among all six healthy individuals and a ROM surface area of 13571 ± 308 deg^2^ for the subject with frozen shoulder. All three sections of the deltoid show greater EMG activity at higher elevation angles. Using such tools enables individuals, surgeons and physiotherapists to measure the maximum envelope of motion in conjunction with muscle activity in order to provide an objective assessment of shoulder performance in the voluntary 3D workspace.

**Graphical abstract:**
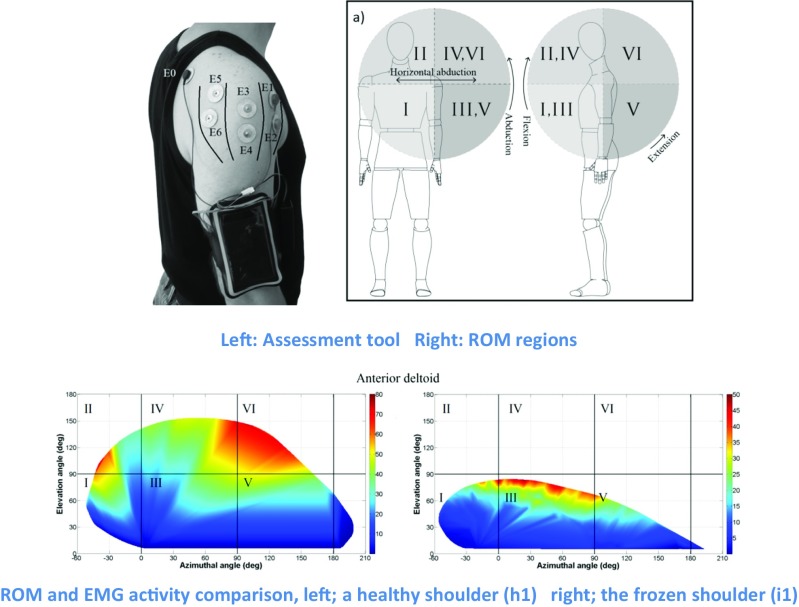
The aim of this study is to develop and evaluate a single IMU combined with an electromyography (EMG) sensor to monitor the 3D reachable workspace with simultaneous measurement of deltoid muscle activity across the shoulder ROM. The assessment tool consists of an IMU sensor, an EMG sensor, a microcontroller and a Bluetooth module. The assessment tool was attached to subjects arm. Individuals were instructed to move their arms with the elbow fully extended. They were then asked to provide the maximal voluntary elevation envelope of the arm in 3D space in multiple attempts starting from a small movement envelope going to the biggest possible in four consecutive circuits. The results showed that there was an average ROM surface area of 27291 ± 538 deg2 among all six healthy individuals and a ROM surface area of 13571 ± 308 deg2 for the subject with frozen shoulder. All three sections of the deltoid show greater EMG activity at higher elevation angles. Using such tools enables individuals, surgeons and physiotherapists to measure the maximum envelope of motion in conjunction with muscle activity in order to provide an objective assessment of shoulder performance in the voluntary 3D workspace.

The aim of this study is to develop and evaluate a single IMU combined with an electromyography (EMG) sensor to monitor the 3D reachable workspace with simultaneous measurement of deltoid muscle activity across the shoulder ROM. The assessment tool consists of an IMU sensor, an EMG sensor, a microcontroller and a Bluetooth module. The assessment tool was attached to subjects arm. Individuals were instructed to move their arms with the elbow fully extended. They were then asked to provide the maximal voluntary elevation envelope of the arm in 3D space in multiple attempts starting from a small movement envelope going to the biggest possible in four consecutive circuits. The results showed that there was an average ROM surface area of 27291 ± 538 deg2 among all six healthy individuals and a ROM surface area of 13571 ± 308 deg2 for the subject with frozen shoulder. All three sections of the deltoid show greater EMG activity at higher elevation angles. Using such tools enables individuals, surgeons and physiotherapists to measure the maximum envelope of motion in conjunction with muscle activity in order to provide an objective assessment of shoulder performance in the voluntary 3D workspace.

## Introduction

The shoulder, and more specifically the glenohumeral joint, provides the largest range of motion in the human body. A healthy shoulder is expected to provide a certain amount of pain-free motion and strength. Shoulder disorders are the third most common location for a musculoskeletal problem, after knee and hip disorders [1]. Most common shoulder disorders can be divided into soft tissue disorders, articular injury or instability, and arthritis causing pain and motion loss leading to difficulties in performing daily activities [2–4].

Shoulder performance can be assessed objectively using different criteria such as the shoulder range of motion (ROM) and electromyography (EMG) at the shoulder muscles. Shoulder performance can also be assessed by clinicians utilising questionnaires, such as the Disabilities of the Arm, Shoulder and Hand Questionnaire (DASH) [1]; Shoulder Pain and Disability Index (SPADI) [5]; Simple Shoulder Test (SST) [6]; Oxford Shoulder Score (OSS) [7]; and the Shoulder Disability Questionnaire (SDQ) [8].

Different measurement tools can be used to analyse human movement. Traditionally, these devices work using one of optical, mechanical, magnetic, structured light or acoustic techniques; however, for measurements of shoulder range of motion in different planes, the most common measurement tools are either mechanical systems used by operators manually or optical devices. Mechanical measurement tools, such as the goniometer [9], inclinometer and plurimeter [5], rely on trained operators and have low accuracy and reliability, while vision-based systems, using optical reflective markers attached to the subject’s limb to be tracked in 3D space, are relatively expensive and time consuming to use due to the experimental setup for each subject [10, 11]. Recent studies [12, 13] have suggested the use of Kinect measurements as a better solution in terms of cost and availability for shoulder ROM tracking. Recently, low-cost wearable inertial measurement unit sensors have overcome many of the limitations of traditional motion tracking systems [14, 15]. These sensors include 3-axis accelerometers (measuring linear acceleration), 3-axis gyroscopes (measuring angular velocity) and a 3-axis magnetometer (measuring magnetic north to compensate for orientation drift). These sensors in combination lead to a more accurate dynamic orientation calculation.

**Fig. 1:**
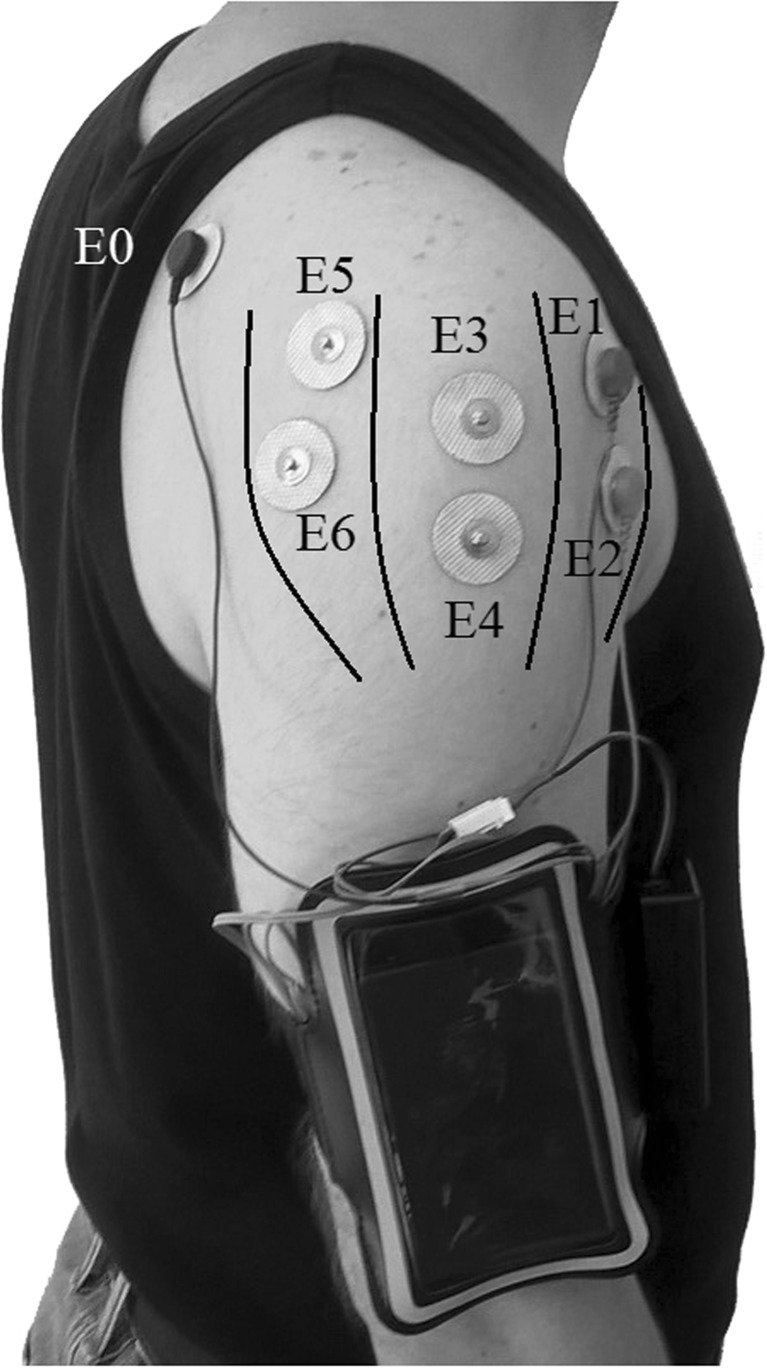
Deltoid sections and surface electrode placement E0, ground; E1/E2, anterior deltoid; E3/E4, middle deltoid; E5/E6, posterior deltoid

The shoulder is comprised of four joints including, glenohumeral, scapulothoracic, sternoclavicular and acromioclavicular. However, the glenohumeral joint has the biggest share in most shoulder motions in daily activities [16, 17]. The deltoid muscle plays an important role as the main shoulder abductor. It consists of three separate sections, known as the anterior deltoid, middle deltoid and posterior deltoid (Fig. 1) [5–7]. It is involved in the majority of shoulder activities, although in different shoulder movements, different deltoid sections are involved in conjunction with other shoulder muscles. The anterior deltoid is more active in flexion, adduction and medial rotation; the middle deltoid has the biggest share in arm abduction among all shoulder muscles; the posterior deltoid provides extension, adduction and lateral rotation [15].

Current systems (using a single IMU) are intended to measure the ROM of the shoulder including all shoulder joint interactions. Most of the studies investigating shoulder ROM measure either passive motion or a specific motion scenario in a specific plane (nonplanar measurement) independently. Haering et al. [18] studied shoulder 3D ROM with all degrees of freedom interactions using a motion analysis system combined with an upper limb kinematic model. In a similar study, Han et al. [19] measured the 3D reachable workspace envelope surface area normalised to subject’s arm length using a stereo camera. However, to study the contribution of each joint individually, a number of IMUs can be attached to different shoulder segments to track their kinematics in real time and 3D space using different shoulder motion analysis protocols [18, 19].

Orientation of objects in 3D space can be described using different forms such as 3 Euler angles, 4-element quaternion vector or a 3 × 3 rotation matrix. Euler angles suffer from a singularity error known as ‘gimbal lock’. Gimbal lock is loss of one degree of freedom in 3D space which causes loss of orientation tracking in higher angles for a short period of time. Both quaternion and rotation matrix techniques do not have any discontinuity across the range of possible 3D orientations, making them the best mathematical algorithm for full tracking of human arm movement in 3D space [14, 20].

There are different IMU sensors and algorithms such as Madgwick and Sabatini to calculate quaternions [21]. Horsak et al. [22] assessed five different IMUs concluding that BNO055 (Bosch Sensortec—BNO055 intelligent 9-axis absolute orientation sensor) is the most suitable in terms of ease of use and data reliability. BNO055 uses a high-speed ARM Cortex-M0 processor and built in fusion algorithms and calibration function [23].

Several studies document EMG activity of shoulder muscles during specific shoulder movement [24]. There are two different types of EMG, intramuscular EMG using needle electrodes inserted into muscles and surface EMG measured with sensors applied to the skin above the muscle belly. Although intramuscular EMG is more reliable in terms of recording actual muscle activity, previous studies have revealed that EMG of the deltoid muscle could be measured accurately using surface electrodes [25].

This study proposes a new strategy and measurement protocol, as well as a novel transducer system, to assess the performance of the shoulder by combining both range of motion and electromyography measurement.

## Methods

### Design of study

In this study, an IMU sensor combined with an EMG sensor to measure the maximum reachable envelope of motion in 3D space with simultaneous collection of deltoid activity is proposed.

Firstly, an assessment tool was designed and developed using a combination of IMU and EMG sensors. Quaternions transmitted from the IMU sensor to a computer are converted into a spherical coordinate system and the accuracy of the IMU sensor is evaluated using a custom joint simulator. The assessment tool was attached to the arms of seven participants as they performed a series of arm movements covering the maximum range of motion they were able to provide. Arm movement and EMG were monitored in real time and recorded for further processing. EMG values were normalised to the Maximum Voluntary Contraction (MVC) recorded by each muscle section during each test [24]. Lastly, the results are discussed for the six healthy shoulder and one frozen shoulder subjects.

### Assessment tool

The assessment tool consists of an IMU sensor (BNO055 intelligent 9-axis absolute orientation sensor), an EMG sensor (MyoWare Muscle Sensor), a microcontroller (ATmega328) and a Bluetooth module (HC-05). The sampled quaternion (calculated using a 32-bit microcontroller running the proprietary BSX3.0 FusionLib software) and raw EMG signals are transmitted to the microcontroller. The raw EMG signal is filtered, rectified and processed according to the root mean square (RMS) procedure by the ATmega microcontroller. The microcontroller then synchronises RMS EMG and IMU sensors data and transmits them to a personal computer through a Bluetooth module at 100 Hz. Software developed in MATLAB (MathWorks Inc., MA, USA) is used to analyse the data in real time and the analysed data is visualised as an animated figure moving its arm. In this way, the performance of the sensor on individuals can be visually inspected during each test. Then, the recorded data is processed and results are presented as graphs. The device weighs 230 g and it is attached to the subject’s arm with an adjustable band in such a way as not to impede movement and so that the subject feels comfortable during the required tests (Fig. 1). This is a relatively low-cost (the overall cost of the system is less than £90), light-weight and portable system developed for ease of use to allow a more subjective assessment of the shoulder.

A quaternion (*q*) is a vector with one real element and three complex elements. Any arbitrary orientation of an object in 3D space can be represented by a unit quaternion as defined below:

1 $$ q=\mathrm{qw}+\mathrm{i}\ \mathrm{qx}+\mathrm{j}\ \mathrm{qy}+\mathrm{k}\ \mathrm{qz} $$

where qw, qx, qy, qz are quaternion elements.

All four quaternion elements are calculated by the microcontroller embedded in BNO055 to be analysed in MATLAB. The quaternion representation can be transformed into a unique rotation matrix using the equation below:

2 $$ R=\left[\begin{array}{ccc}{\mathrm{qw}}^2+{\mathrm{qx}}^2-{\mathrm{qy}}^2-{\mathrm{qz}}^2& 2\times \mathrm{qx}\times \mathrm{qy}-2\times \mathrm{qw}\times \mathrm{qz}& 2\times \mathrm{qx}\times \mathrm{qz}+2\times \mathrm{qw}\times \mathrm{qy}\\ {}2\times \mathrm{qx}\times \mathrm{qy}+2\times \mathrm{qw}\times \mathrm{qz}& {\mathrm{qw}}^2-{\mathrm{qx}}^2+{\mathrm{qy}}^2-{\mathrm{qz}}^2& 2\times \mathrm{qy}\times \mathrm{qz}-2\times \mathrm{qw}\times \mathrm{qx}\\ {}2\times \mathrm{qx}\times \mathrm{qz}-2\times \mathrm{qw}\times \mathrm{qy}& 2\times \mathrm{qy}\times \mathrm{qz}+2\times \mathrm{qw}\times \mathrm{qx}& {\mathrm{qw}}^2-{\mathrm{qx}}^2-{\mathrm{qy}}^2+{\mathrm{qz}}^2\end{array}\right] $$

The rotation matrix of the arm in a neutral position ([*R*_0_]) is considered as a reference. The neutral position is the position of the arm resting naturally at zero degree elevation. The rotation matrix of any arbitrary arm orientation relative to this reference is as follows:

3 $$ \left[R\right]={\left[{R}_0\right]}^{-1}\times \left[R\right] $$

In this study, the aim is to define arm motion using spherical coordinate parameters (azimuthal angle and elevation angle). Spherical coordinates helped to avoid Codman’s paradox [26, 27] by ignoring the axial rotation of the arm around the long humerus axis. This was achieved by defining a Cartesian coordinate system using the rotation matrix and then converting it into spherical coordinates.

**Fig. 2:**
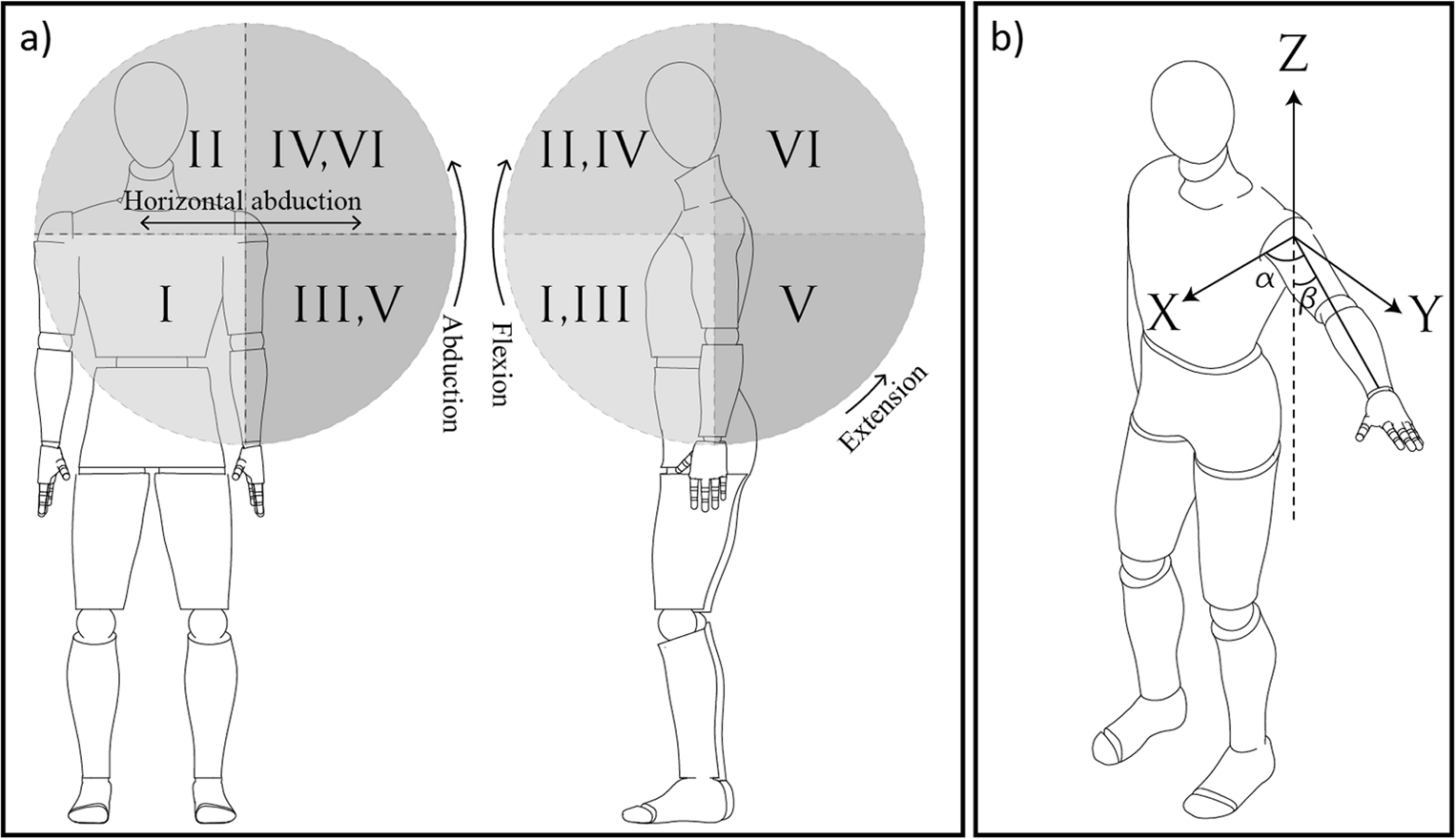
**a** ROM regions. **b** Arm spherical coordinates where α represents azimuthal angle and β is the elevation angle

Graphical visualisation of 3D ROM regions is shown in Fig. 2a and regions are described in Table 1.

**Table 1:** ROM regions in spherical coordinate

Region	Shoulder motion
I	Lower medial elevation
II	Higher medial elevation
III	Lower lateral elevation
IV	Higher lateral elevation
V	Lower posterior elevation
VI	Higher posterior elevation

The origin of the Cartesian coordinate system ([*x*_*o*_, *y*_*o*_, *z*_*o*_]) is defined at the shoulder joint when the arm is in its neutral position and it is located at the centre of rotation (COR) of the arm as shown in Fig. 2b. The coordinate of an arbitrary point on the arm having distance of *r* from the origin is defined as [*x*_*n*_, *y*_*n*_, *z*_*n*_]. While the arm moves in 3D space, the new coordinate of this arbitrary point is calculated using:

4 $$ \left[{x}_n,{y}_n,{z}_n\right]={\left[R\right]}^{\ast}\left[{x}_o,{y}_o,{z}_o\right] $$

In this study, rotation around the *z*-axis (azimuthal angle) of the shoulder is considered as horizontal abduction and rotation around the *x*-axis (elevation angle) as abduction. Hence, the Cartesian coordinate of the moving arm can be transformed to the spherical coordinates using the equations below:

5 $$ r=\sqrt{x_n^2+{y}_n^2+{z}_n^2} $$

6 $$ \beta ={\mathit{\cos}}^{-1}\left(\frac{z_n}{r}\right) $$

7 $$ \alpha ={\mathit{\tan}}^{-1}\left(\frac{y_n}{x_n}\right) $$

### IMU performance assessment

A gimbal test stand was manufactured to quantify the IMU sensor performance and compare its calculations against known angle rotations. The gimbal is able to provide full pitch and yaw motion using a pair of servomotors (HS-7950TH—Hitec RCD USA, Inc.). The IMU sensor is placed on the gimbal test stand and initial orientation was recorded as the arm orientation in the rest condition.

To evaluate accuracy and repeatability of the IMU sensor, full arm elevation in different abduction planes as well as horizontal abduction is simulated by the gimbal mechanism (each test was repeated three times). Input angles provided by servo motors are compared to measured spherical coordinate angles by the IMU. A maximum error of 3^o^ for elevation angle and maximum error of 2^o^ for azimuthal angle were recorded during the tests. The results showed the validity of the sensor performance since they are comparable with precise rotation angles provided by servo motors [28].

### Subjects

Six volunteer subjects with healthy shoulders (four men, two women) with average age of 27.3 ± 3.4 years, average height of 173 ± 6 cm and average weight of 73 ± 8 kg and one male participant with a frozen shoulder (age 42, height 176 cm and weight of 75 kg) were studied. None of the subjects with healthy shoulders reported a history of shoulder injury, pain or instability. The study was approved by the research ethics committee of Bournemouth University. All subjects gave their written informed consent before inclusion in the study.

### Experimental procedures

Prior to pre-gelled electrode placement, the skin on the shoulder was shaved and cleaned using alcohol [29, 30]. Six disposable surface electrodes were placed over the muscle belly by visual inspection and palpation of the muscle sections parallel to the muscle fibre direction, with a centre to centre distance of 35 mm [31]. Electrodes for recording the anterior deltoid were placed 25 mm below the anterior crest of the acromion, electrodes for the middle deltoid were located halfway between the acromion and the deltoid tubercle and electrodes for the posterior deltoid were positioned 25 mm below the posterior crest of the acromion. The reference electrode was positioned over the scapula [24, 32–34]. Electromyography of all three sections of the deltoid was evaluated in response to shoulder elevation in 3D space.

The subjects stood in a stationary position facing the same direction during the experiment. Two practice motions were performed before each test. The subjects were verbally instructed to move their arm as far as they can in all directions at their own comfortable speed.

The assessment tool was attached to the subjects arm. Individuals were instructed to move their arms with the elbow fully extended. They were then asked to provide the maximal voluntary elevation envelope of the arm in 3D space in multiple attempts starting from a small movement envelope going to the biggest possible in four consecutive circuits. Each subject was asked to start their arm elevation medially, then anteriorly, cranially, posteriorly, laterally and then back to the initial rest position.

A demonstrator performed the movements in front of the subject to show the order of movements while asking the subject to provide their maximal voluntary elevation. Participants were advised not to move their legs and chest and to keep their torso facing the same direction throughout the movement. To evaluate the repeatability of each test, each subject performed the test three times. EMG of muscles was recorded simultaneously with arm motions from each of the three deltoid sections sequentially.

**Fig. 3:**
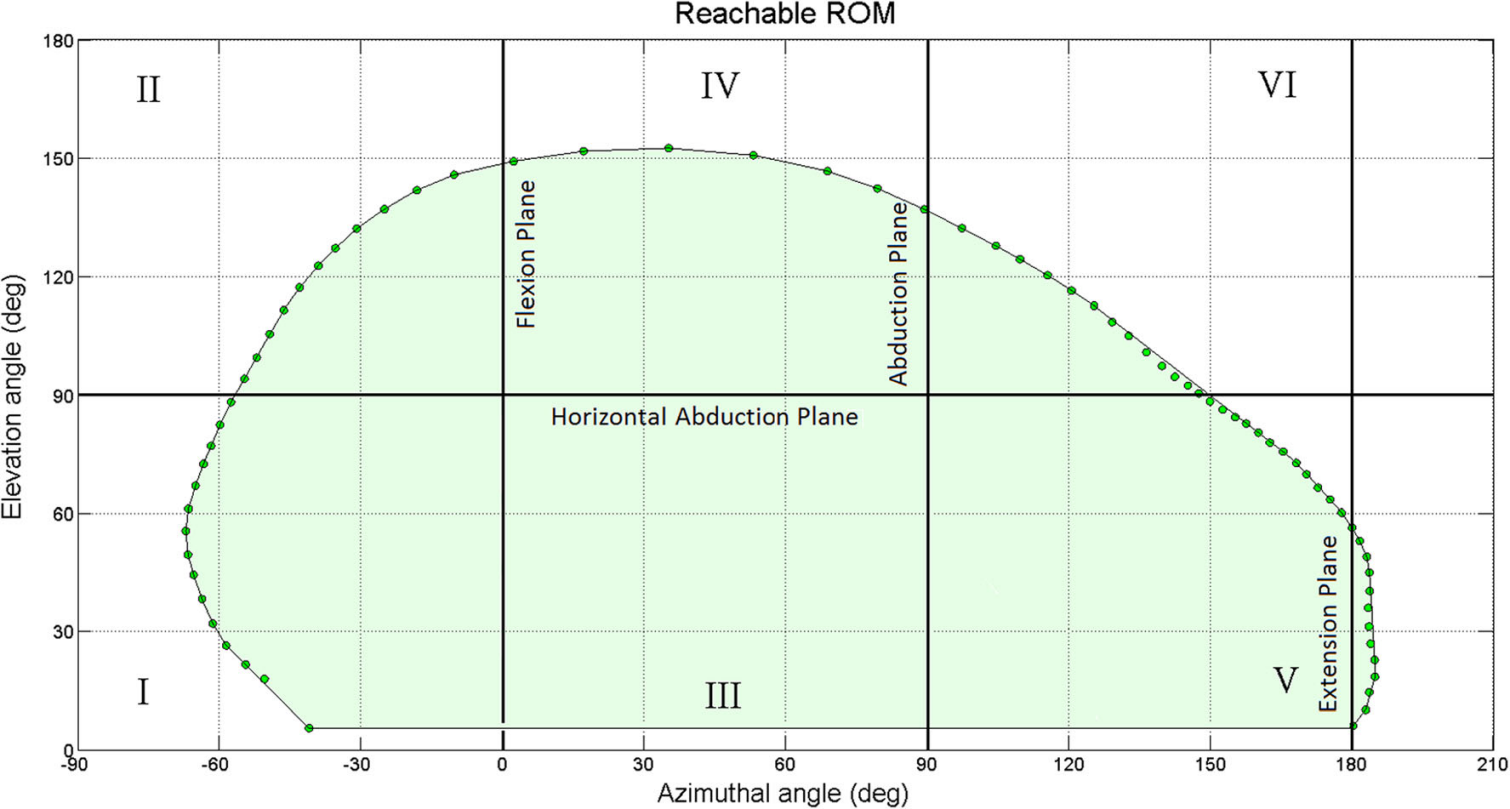
Sample ROM data collection

In the case that any extra body movements such as bending or trunk rotation were observed by the demonstrator, the test was repeated. In all three tests, subjects were informed that comfortable axial rotation could be utilised if necessary. As spherical coordinates are used in this study, only two angles of azimuthal (α) and elevation (β) are considered while rotation of the humerus around its axis is ignored. An example of reachable workspace in spherical coordinate is shown Fig. 3.

## Results

Azimuthal angle versus elevation angle of the arm movement in 3D space are plotted while EMG intensity of any arm position is presented by colour contours to quantify maximum reachable surface area of shoulder, maximum shoulder elevation in different planes separately and EMG activity of each section of the deltoid at any arbitrary orientation of shoulder. Then, the results within six healthy shoulders and one frozen shoulder are compared.

Each subject repeated the same test three times and although envelope profiles are slightly different, a maximum variation coefficient of 8.3% was found across all subjects. An average ROM, maximum elevation of each individual’s arm in different planes, the average surface area as well as maximum values from the mean of six shoulders and the one frozen shoulder showed a significant difference. Results are compared in Table 2. ROM surface area of 27291 ± 538 deg^2^ was found with a variation coefficient of 5% among all six healthy individuals. The subject suffering from a frozen shoulder was able to provide only 13571 ± 308 deg^2^ ROM showing a 67% difference from the average of healthy shoulders. Results are presented in Table 2.

**Table 2:** ROM measurements

	Average surface area (deg^2^)	Coefficient of variation	Max Flexion	Max Abduction	Max extension	Max horizontal abduction	Anterior deltoid EMG (%MVC)	Middle deltoid EMG (%MVC)	Posterior deltoid EMG (%MVC)
h1	27732	4.2	150	153	65	213	72	68	49
h2	26590	3.9	140	131	42	200	45	51	28
h3	26844	3.2	146	137	57	190	52	53	31
h4	27122	5	162	160	55	190	74	43	34
h5	28002	8.3	150	124	60	205	71	61	49
h6	27458	5.3	148	142	51	203	49	63	29
Healthy Mean	27291	5	149 ± 7	141 ± 13	55 ± 8	200 ± 9	60 ± 13	57 ± 9	37 ± 10
i1	13571	3	86	64	NA	NA	35	30	15

*h* healthy shoulders, *i* injured shoulder

**Fig. 4:**
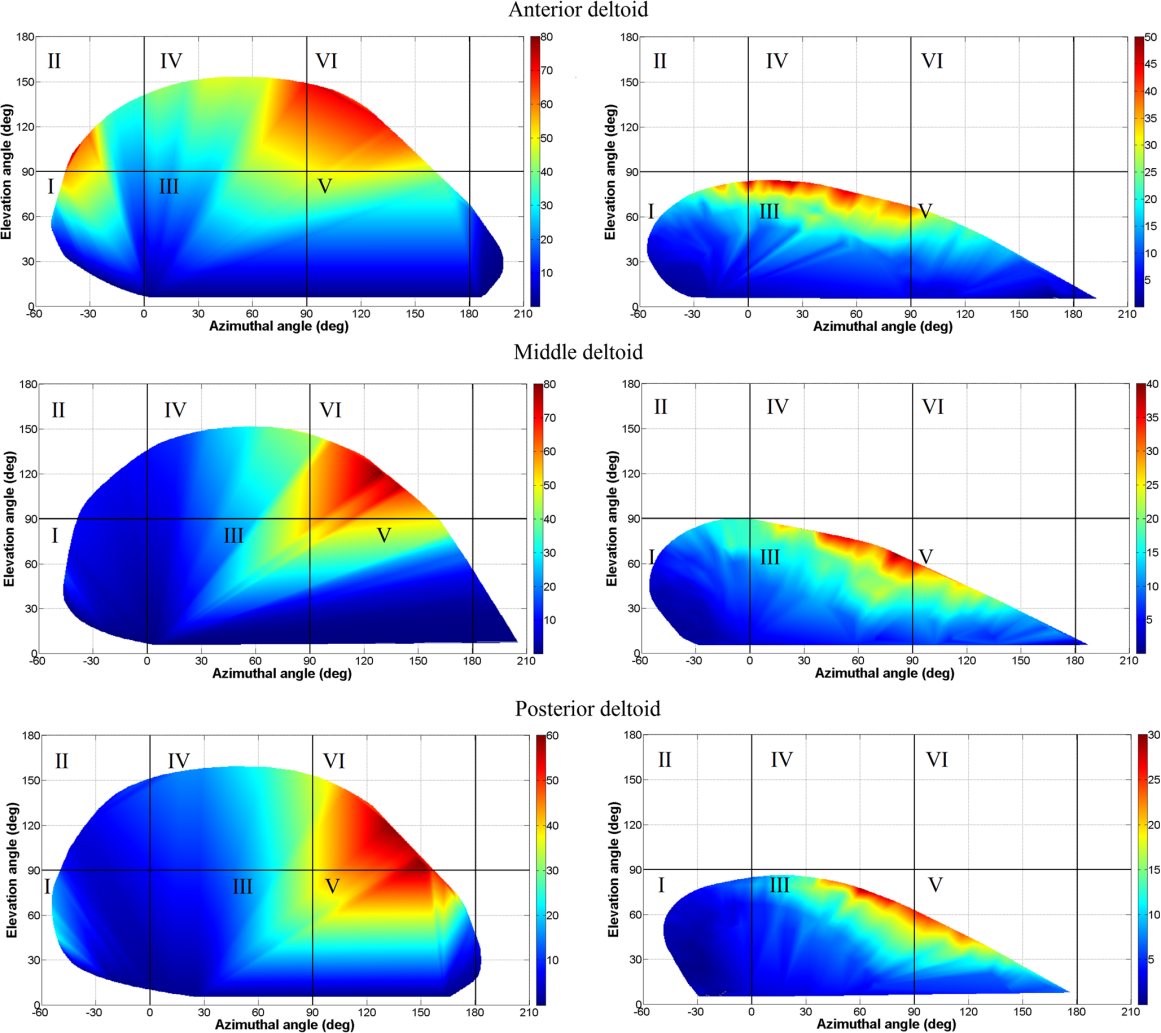
ROM and EMG activity comparison. Left, a healthy shoulder (h1); right, the frozen shoulder (i1)

As shown in Fig. 4, the healthy shoulder was able to cover the majority of regions I, III and IV, which are where most daily activities are performed. According to the graphs, the healthy shoulder shows the highest EMG activity at high posterior elevation angles (region VI). For the frozen shoulder, the highest EMG activity occurs at low lateral elevation (region III).

It is also observed that the anterior deltoid is the most active muscle at higher elevation angles (regions II, IV and VI). The anterior deltoid showed the largest recorded EMG among all sections with average of 60 ± 13%. Although the middle and posterior deltoid showed very similar patterns of EMG activity, the average EMG of the middle deltoid (57 ± 9%) is higher than that of the posterior deltoid (37 ± 10%).

## Discussion

There are different methods to evaluate shoulder performance in terms of pain-free motion, manoeuvrability, strength and muscle activity. To measure ROM at the shoulder, there are different methods, protocols and tools mentioned in the literature. Most protocols study shoulder ROM in a single plane of motion. However, in this study, using an IMU sensor to measure the maximum envelope of motion in 3D space is proposed. Using an EMG sensor combined with the IMU aids in the evaluation of muscle activity of deltoid sections.

All the subjects performed the requested arm movement with an extended elbow, using comfortable arbitrary axial rotation when needed. Each test was performed three times and EMG of one section of the deltoid was recorded in each test. Results are represented in graphs which gives figures for both EMG and ROM.

In terms of reachable surface area, the subjects showed a maximum variation coefficient of 8.3% across three tests. A coefficient of variation of 5% was observed between all six healthy shoulders. The subject with the frozen shoulder showed 13571 deg^2^ which was only 67% of the average of healthy shoulders. Measured maximum values in separate planes are in agreement with the values from literature where maximum values are measured in separate single planes [35–37].

In terms of EMG, the anterior deltoid was the most active muscle at higher elevations. It also showed the largest average EMG activity of 60 ± 13%. The EMG activity of both the middle and posterior deltoids occurred in the same regions; however, the activity of the middle deltoid was greater; 57 ± 9% compared to the 37 ± 10% of the posterior deltoid. Each deltoid within the healthy shoulder showed greater EMG activity compared to the corresponding deltoid in the frozen shoulder. The lower EMG activity of the deltoid sections in the frozen shoulder could be due to the limited range of motion of the arm.

Currently, there is no similar study assessing full 3D ROM of the shoulder associated with muscle activity; however, there are some studies evaluating shoulder ROM in different planes of motion and some studies evaluating deltoid muscle activity in different shoulder activities [22–24, 32, 35].

### Limitations

In this study, arm movement is considered while the body is stationary. Both glenohumeral and scapular joint contributed to the arm movement, although using one IMU attached to the subjects arm does not allow the scapula rhythm as well as torso movements involved in each subject experimental performance to be differentiated. Adding two more IMUs, one on the thorax and one attached to the scapula, enables the investigation of the effect of scapula rhythm of individuals as well an improved means of detecting if the subject moves their body to reach the maximum ROM. In this study, interaction of all shoulder joints is simplified as a spherical joint moving in 3D space while its motion is described by spherical coordinate angles (elevation and azimuthal angles).

As mentioned before, EMG values were normalised to the Maximum Voluntary Contraction recorded by each muscle section during each test. However, it should be noted that using MVC as the reference for normalising EMG data might not accurately show maximum muscle activation capacity in the muscle in all individuals [38].

## Conclusion

Shoulder disorders such as rotator cuff deficiency or glenohumeral/acromioclavicular joint problems, where the shoulder shows limited range of motion, may be assessed in terms of three-dimensional ROM surface area and with EMG. It may also be used to quantify and monitor progress of a rehabilitation program.

It has been shown that using a wearable IMU to track human motion is possible without the need for complex camera-based tracking systems or mechanical measurement tools which suffer from inaccuracies. The IMU sensor was attached to six healthy shoulders and one impaired frozen shoulder and results are compared. At the same time, EMG activity of the subjects during 3D movements was monitored and compared for each of the anterior deltoid, middle deltoid and posterior deltoid.

The data provides information on the shoulder range of motion in specific standard planes such as abduction, flexion as well as any point of interest in the whole 3D range of motion. It also provides information on the relative magnitude of EMG data in each section of deltoid across the whole range of motion.

EMG of the shoulder shows that in all cases, all three sections of deltoid were highly active at higher elevation. A prominent feature is that a significantly higher EMG is observed in region II, IV and VI in healthy shoulders and I, III and VI in the frozen shoulder.

The minimal setup time needed for the sensor and relatively low cost has the potential to make the proposed system a practical assessment tool for individuals, surgeons and physiotherapist for objective assessment of shoulder motion as well as muscle EMG monitoring. Future work will include the combination of more IMU sensors mounted on the scapula and torso to track the whole upper body movement while moving the arm in 3D space. The future system will also include multiple EMG sensors on the deltoid and other shoulder muscles such as lower and upper trapezoid. However, this preliminary study provided proof of concept.
